# Correction: Enhancing the anti-ovarian cancer activity of quercetin using a self-assembling micelle and thermosensitive hydrogel drug delivery system

**DOI:** 10.1039/c9ra90071c

**Published:** 2019-10-16

**Authors:** Guangya Xu, Bin Li, Ting Wang, Jun Wan, Yan Zhang, Jingwei Huang, Yangmei Shen

**Affiliations:** Department of Anatomical Pathology and Pathophysiology, College of Medicine, Chengdu University Chengdu People’s Republic of China; Department of Pathology, West China Second University Hospital, Sichuan University Chengdu 610041 PR China symjulia@126.com +86 2885164060 +86 2885164063; Key Laboratory of Birth Defects and Related Diseases of Women and Children (Sichuan University), Ministry of Education, West China Second University Hospital, Sichuan University Chengdu 610041 PR China

## Abstract

Correction for ‘Enhancing the anti-ovarian cancer activity of quercetin using a self-assembling micelle and thermosensitive hydrogel drug delivery system’ by Guangya Xu *et al.*, *RSC Adv.*, 2018, **8**, 21229–21242.

The authors regret that Fig. 8A and C in the original article contained errors, due to incorrect data sets being used for the image preparation. The correct version of Fig. 8 is shown below.

**Fig. 1:**
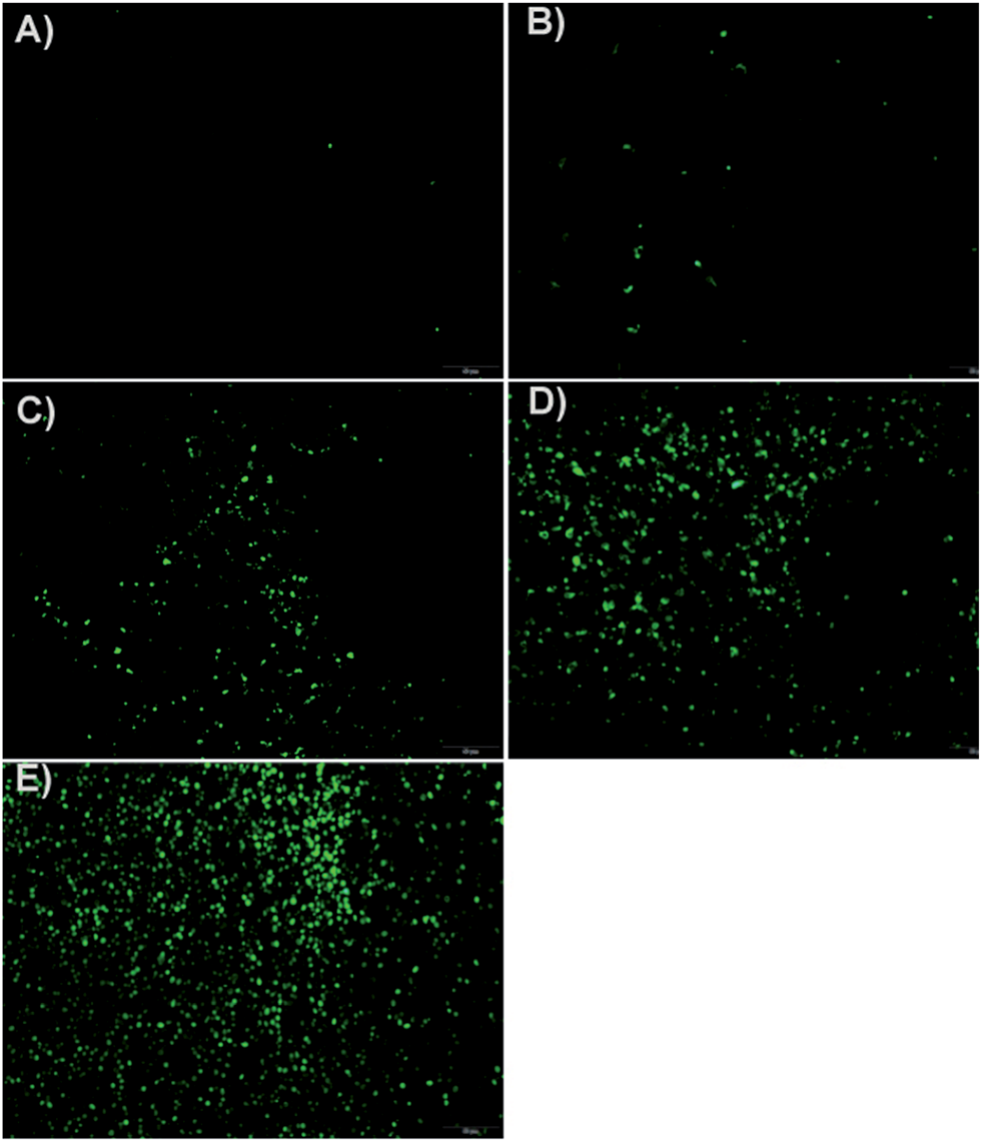
Tunnel assay. The tumor tissue sections of the normal saline (NS) treated group (A), empty hydrogel (EG) treated group (B), free quercetin (FQ) treated group (C), Qu-M (QM) treated group (D), and Qu-M–hydrogel composite (QMG) treated group (E) were stained with Tunnel for the cell apoptosis assay, indicating that inducing apoptosis may be one of the anti-tumor mechanisms of the Qu-M–hydrogel composites (QMGs), Qu-M (QM), and free quercetin (FQ) *in vivo*.

In addition, on page 21237 of the original manuscript in the section titled “3.3.4 Induction of tumor cell apoptosis *in vivo*”, a sentence should be corrected. “The apoptotic index in Qu-M–hydrogel composites, Qu-M, free quercetin (Free-Qu), empty hydrogel and normal saline (NS) were 72.7% ± 6.34%, 43.23% ± 4.68%, 28.23% ± 3.23%, 2.14% ± 0.57%, and 1.31% ± 0.43, respectively,” should be “The apoptotic index in Qu-M–hydrogel composites, Qu-M, free quercetin (Free-Qu), empty hydrogel and normal saline (NS) were 72.7% ± 6.34%, 43.23% ± 4.68%, 23.41% ± 5.37%, 2.14% ± 0.57%, and 1.52% ± 0.35, respectively”.

The Royal Society of Chemistry apologises for these errors and any consequent inconvenience to authors and readers.

## Supplementary Material

